# Neurofilament Light Chain: A Potential Biomarker for Chemotherapy-Induced Peripheral Neuropathy in Pediatric and Adolescent Young Adults with Leukemia or Lymphoma

**DOI:** 10.3390/cancers18172756

**Published:** 2026-08-25

**Authors:** Jennifer A. Belsky, Allie Carter, Michael E. Roth, Audrey Leisinger, Etan Orgel, AnnaLynn M. Williams, Rozalyn L. Rodwin, Bryan P. Schneider, Ellen M. Lavoie Smith

**Affiliations:** 1 Department of Pediatrics, Indiana University School of Medicine, Indianapolis, IN 46202, USA; 2 Division of Pediatric Hematology, Oncology, and Stem Cell Transplant, Riley Hospital for Children, Indianapolis, IN 46202, USA; aleisinger@iuhealth.org; 3 Department of Biostatistics and Health Data Science, Indiana University School of Medicine, Indianapolis, IN 46202, USA; alternet@iu.edu; 4 Division of Pediatrics, MD Anderson Cancer Center, Houston, TX 77030, USA; mroth1@mdanderson.org; 5 Cancer and Blood Disease Institute, Children’s Hospital Los Angeles, Los Angeles, CA 90027, USA; eorgel@chla.usc.edu; 6 Department of Pediatrics, Keck School of Medicine, Los Angeles, CA 90033, USA; 7 Department of Surgery, Division of Supportive Care in Cancer, James P. Wilmot Cancer Institute, University of Rochester Medical Center, Rochester, NY 14642, USA; annalynn_williams@urmc.rochester.edu; 8 Department of Pediatrics, Yale School of Medicine, New Haven, CT 06510, USA; rozalyn.rodwin@yale.edu; 9 Yale Cancer Center, Yale School of Medicine, New Haven, CT 06510, USA; 10 Department of Medicine, Indiana University School of Medicine, Indianapolis, IN 46202, USA; bpschnei@iu.edu; 11 School of Nursing, University of Alabama at Birmingham, Birmingham, AL 35294, USA; esmith3@uab.edu

**Keywords:** chemotherapy-induced peripheral neuropathy, pediatric cancer, supportive care, biomarker

## Abstract

**Introduction**: Chemotherapy-induced peripheral neuropathy (CIPN) is a common and dose-limiting toxicity in child, adolescent, and young adult (CAYA) oncology populations. Despite its clinical impact, objective biomarkers for early detection and monitoring remain limited. Neurofilament light chain (NfL), a marker of axonal injury, has emerged as a potential circulating biomarker of CIPN in adults and potentially for CAYAs. This pilot study evaluates the association between NfL and patient-reported CIPN severity in CAYAs. **Methods**: We conducted a prospective pilot study of 26 patients with acute lymphoblastic leukemia or lymphoma. CIPN was assessed using FACT-GOG/NTx scores. Linear mixed-effects models evaluated associations between NfL and neuropathy over time, adjusting for age and time from baseline. Logistic mixed models assessed the relationship between NfL and clinically significant neuropathy (FACT-GOG/NTx ≤ 40). **Results**: NfL was significantly associated with worsening neuropathy. A 50-unit increase in NfL corresponded to a 1.1-point decrease in FACT-GOG/NTx score (*p* < 0.001). Each doubling of NfL was associated with a 0.61-point decrease in FACT-GOG/NTx (*p* < 0.001). Higher NfL levels increased odds of neuropathy (OR 4.62, *p* < 0.001). Associations were strongest in leukemia patients and not observed in Hodgkin lymphoma when separately analyzed. **Conclusions**: This pilot study demonstrates that circulating NfL correlates with patient-reported neuropathy severity, supporting its role as a potential biomarker for CIPN in CAYAs. Differences between leukemia and lymphoma cohorts may reflect treatment-specific neurotoxicity patterns and should be validated in larger prospective studies. Limitations include small sample size, heterogeneity, and limited power for subgroup analyses. If validated, NfL could be incorporated into routine toxicity monitoring to identify patients at highest risk for progressive CIPN, enabling earlier supportive care interventions, referral to rehabilitation services, or enrollment in biomarker-guided prevention and treatment trials before irreversible nerve injury occurs.

## Simple Summary

Chemotherapy-induced peripheral neuropathy is a common side effect of cancer treatment that can cause numbness, tingling, weakness, pain, and difficulty performing everyday activities. In children, adolescents, and young adults, these symptoms can interfere with treatment, reduce quality of life, and persist long after therapy ends. Unfortunately, clinicians currently rely largely on patient-reported symptoms to recognize neuropathy, and there are no widely used blood tests to detect nerve injury early. This study evaluated whether neurofilament light chain, a protein released into the bloodstream after nerve damage, is associated with the severity of neuropathy symptoms during cancer treatment. We found that higher neurofilament light chain levels were associated with worse patient-reported neuropathy, supporting its potential as an objective biomarker of chemotherapy-related nerve injury. If validated in larger studies, this biomarker could help identify patients at risk earlier, improve monitoring during treatment, and support the development of strategies to prevent or reduce nerve damage.

## 1. Introduction

Chemotherapy-induced peripheral neuropathy (CIPN) is a frequent and clinically significant toxicity affecting children, adolescents, and young adults (CAYAs) undergoing cancer treatment [1]. Hallmarks of CIPN include a spectrum of sensory, motor, and autonomic symptoms, including numbness, tingling, neuropathic pain, distal weakness, impaired reflexes, gait instability, and gastrointestinal dysfunction [1]. Symptoms of CIPN often emerge during therapy and persist long after treatment completion, contributing to lasting impairments in physical functioning, academic performance, and overall health-related quality of life [2]. Research has demonstrated a substantial decrease in quality of life associated with CIPN, with differences approaching large effect sizes when compared to patients without neuropathy. Vincristine is a cornerstone of therapy for many pediatric and AYA malignancies; however, its use is frequently complicated by CIPN, which can result in sensory, motor, and autonomic symptoms and may necessitate dose reduction or treatment interruption. Preclinical detection of CIPN is essential, as timely identification may allow for interventions that mitigate progression and reduce long-term morbidity [3]. However, current approaches to early detection are lacking in both children and AYAs [4]. Although the incidence of CIPN is high depending on treatment exposures and CIPN assessment methods, it is frequently underrecognized in routine clinical care [4]. Many patients are not identified as having neuropathy until symptoms are severe or newly identified following completion of therapy [5]. In a study of children with leukemia who received neurotoxic chemotherapy, 38% of children and AYA cancer survivors experienced CIPN, yet CIPN was not discovered until after therapy had concluded in 82% of cases [5]. The use of suboptimal CIPN measures is partly attributable to limitations of commonly available assessment tools. The Common Terminology Criteria for Adverse Events (CTCAE), the standard clinician-reported grading system, relies heavily on subjective judgment and lacks sensitivity for early or mild symptoms, particularly in pediatric populations [6]. As a result, CIPN is often documented only after it becomes clinically significant, at which point the only recommended treatment is chemotherapy dose reduction or omission of the neurotoxic chemotherapeutic agent.

Patient-reported outcome (PRO) measures, including instruments such as the Functional Assessment of Cancer Therapy/Gynecologic Oncology Group—Neurotoxicity (FACT-GOG/NTx) and EORTC CIPN20, provide a more sensitive approach to capturing neuropathy symptoms and have demonstrated validity and reliability in pediatric oncology populations [7,8]. However, neither instrument has been formally validated in children younger than 5 years of age, despite this age group representing a key population at risk for CIPN because of the widespread use of vincristine-containing treatment regimens. These tools improve detection reliability and sensitivity compared to clinician grading and better reflect patient experience. However, PROs remain inherently subjective and are limited to identifying neuropathy only after symptom onset [9,10,11]. In addition, reliance on symptom-based measures introduces challenges in clinical trials, including response variability and placebo effects, which may complicate interpretation of intervention efficacy.

There is a critical need for objective biomarkers that can detect neurotoxicity earlier in the clinical course and provide complementary information to clinical and objective CIPN assessments. Neurofilament light chain (NfL), a biomarker of axonal damage, has recently emerged as a promising potential candidate for CIPN detection [12]. NfL is a structural protein highly expressed in neuronal axons that is released into the circulation following neuroaxonal injury. Because CIPN is characterized by chemotherapy-induced axonal damage, increasing circulating NfL may provide an objective measure of ongoing peripheral nerve injury, with prior studies demonstrating associations between NfL concentrations and CIPN severity [13]. NfL is released into circulation following neuronal damage and can be measured in plasma, offering a minimally invasive approach to quantifying axonal injury. In the context of neurotoxic chemotherapy, elevations in NfL may reflect ongoing nerve damage preceding or accompanying clinical symptoms, thereby providing a potential tool for early detection and real-time monitoring of CIPN [14,15].

While interest in NfL as a biomarker of CIPN is growing, most available data are derived from adult populations, and studies in pediatric and AYA patients remain limited to small pilot data [16,17,18]. Consequently, a significant gap persists in understanding how NfL relates to neuropathy severity and symptom burden in younger patients receiving chemotherapy. To address this gap, we conducted a prospective pilot study to evaluate longitudinal changes in NfL and examine its association with validated PRO scores in children and AYAs undergoing treatment with neurotoxic agents.

## 2. Materials and Methods

### 2.1. Study Design

This single-center, prospective study examined children and AYAs with acute lymphoblastic leukemia (ALL), Hodgkin lymphoma (HL), or non-Hodgkin lymphoma (NHL) who received a chemotherapy regimen that included a neurotoxic chemotherapy agent. Patients were eligible if they (1) were between the ages of ≥2–26 years; (2) were diagnosed with ALL, HL, or NHL; (3) received a neurotoxic chemotherapy agent, including vincristine, vinblastine, and/or brentuximab vedotin; and (4) received chemotherapy at Riley Hospital for Children. Enrollment must have been completed <5 days prior to receiving the first dose of a neurotoxic agent. Patients with pre-existing neuropathy were excluded. The study was approved by the Indiana University Institutional Review Board and conducted in accordance with the Declaration of Helsinki.

CIPN was assessed using the Functional Assessment of Cancer Therapy/Gynecologic Oncology Group—Neurotoxicity (FACT-GOG/NTx) questionnaire, a validated patient-reported outcome measure used to assess the severity of chemotherapy-induced peripheral neuropathy in adolescents and young adults. The FACT-GOG/NTx includes 11 items evaluating sensory, motor, hearing, and functional symptoms, with total scores ranging from 0 to 44; higher scores indicate fewer neuropathy symptoms [8,19]. Patients with newly diagnosed ALL received therapy according to Children’s Oncology Group (COG) protocols AALL1731, AALL1732, or AALL0434, patients with newly diagnosed HL according to COG/Alliance S1826, and patients with newly diagnosed NHL according to COG ANHL1131 [20,21,22,23,24]. Dosing and vinca alkaloid exposure can be found in Supplemental Table S1. Blood samples (6 mL) and the FACT-GOG/NTx were collected at each study timepoint. Study timepoints varied by disease type and included a baseline blood sample (obtained within 5 days before initiation of chemotherapy), day 1 of each subsequent chemotherapy cycle (approximately every 4–8 weeks), end of therapy (defined as 6–12 weeks after the last chemotherapy dose), and every 3 months thereafter (±3 months) until resolution of CIPN symptoms on the FACT-GOG/NTx.

All blood samples were processed by clinical research office staff and stored as part of the Indiana Biobank according to standard procedure. Plasma biomarker assays are run in the National Centralized Repository for Alzheimer’s Disease and Related Dementias (NCRAD) Biomarker Assay Laboratory (BAL). Each assay plate or run is balanced on age, gender, and collection visit for longitudinal samples. Plasma samples are centrifuged at 10,000× *g* for 5 min prior to analysis. Each sample run on the Quanterix is processed in duplicate, starting with dilution with a specific diluent kit per the manufacturer’s instructions on a Tecan Fluent 1080 automated liquid handler (Tecan Group Ltd., Männedorf, Switzerland). Plasma was analyzed on a Quanterix Simoa HD-X. NfL was assessed using the Simoa^®^ HD-X Analyzer (Quanterix Corporation, Billerica, MA, USA) with the Simoa^®^ NF-Light Advantage PLUS kit (Quanterix Corporation, Billerica, MA, USA). Manufacturer-provided quality control (QC) samples are on each plate to verify that results meet quality criteria for analytical performance provided by the manufacturer as well as lab-specific expectations. Pooled plasma reference (PPR) samples are analyzed on each plate to monitor assay performance in the laboratory. Lot-to-lot bridging is performed by NCRAD BAL (Indianapolis, IN, USA) for all Quanterix assays to ensure comparability between studies by detecting and controlling for changes in kit performance due to lot.

### 2.2. Statistical Analysis

Participant characteristics were summarized using frequencies and percentages for categorical variables and medians with interquartile ranges for continuous variables. Clinical assessment timepoints were defined separately for ALL, NHL, and HL cohorts based on treatment phases, and FACT-GOG/NTx scores were summarized across these timepoints.

Univariable linear mixed-effect models evaluated the association between individual demographic and clinical variables and FACT-GOG/NTx scores over time, adjusting for days from baseline. Variables with a significant association with FACT-GOG/NTx scores were included as covariates in the final models. Given potential skewness in both the outcome (FACT-GOG/NTx score) and main predictor (sNfL), model assumptions were evaluated, and linear models were identified as the best-fitting distributions within the exponential family. Model performance was assessed using marginal and conditional R^2^ values, scaled deviance, and intraclass correlation coefficients (ICCs), and multicollinearity was evaluated using variance inflation factors.

The primary analysis used linear mixed-effects models to examine the association between NfL (pg/mL) and FACT-GOG/NTx scores over time. Preliminary models were run for the effect of NfL on FACT-GOG/NTx score and neuropathy, adjusted for time since baseline only (modeled in 3-month increments). For the main models, fixed effects included time since baseline (modeled in 3-month increments) and age at diagnosis, with a random intercept for each participant to account for repeated measurements. NfL was modeled in two ways: (1) as a continuous variable scaled to a 50-unit increase and (2) as a log2-transformed variable to evaluate the effect of doubling NfL levels. While adjusting for time and age, model fit was comparable between approaches.

A secondary linear mixed-effects model evaluated the association between peak NfL and longitudinal FACT-GOG/NTx scores. Additionally, a mixed-effects logistic regression model was used to assess the association between NfL and clinically significant neuropathy, defined as FACT-GOG/NTx ≤ 40. A FACT-GOG/NTx score ≤40 (a ≥4-point reduction from the maximum score of 44) was selected a priori, based on the literature that has indicated that a 4-point change is the minimally clinically important difference for FACT-GOG/Ntx [25,26,27]. Both additional models used the same covariate structure and random intercept as the primary analysis and included the same fixed effects of time since baseline and age. Sensitivity analyses were conducted by restricting the cohort to participants with predefined study timepoints and by analyzing leukemia and lymphoma subgroups separately using the same modeling framework. Exploratory analyses evaluated the relationship between prior NfL measurements and subsequent FACT-GOG/NTx scores by incorporating lagged NfL values, NfL from the prior visit, into linear mixed-effects models within each disease subgroup.

All analyses were performed using R Version 4.4.1, with statistical significance defined as a two-sided *p*-value < 0.05.

## 3. Results

### 3.1. Patient Characteristics

A total of 26 patients were included in the analysis, with a median age of 15.4 years (IQR 9.4–17.0) (Table 1). The cohort consisted of children and AYAs with acute lymphoblastic leukemia (*n* = 11, 42%) or lymphoma (*n* = 15, 58%) who were treated with a neurotoxic chemotherapy agent. Baseline FACT-GOG/NTx scores were high, with a median score of 44, consistent with minimal to no neuropathy symptoms at treatment initiation. Baseline NfL levels were similarly low, with a median of 6 pg/mL.

### 3.2. FACT-GOG/NTx and NfL Scores over Time

Increasing age was independently associated with worse neuropathy over time as measured by the FACT-GOG/NTx. Each additional year of age was associated with an average 0.25-point decrease in FACT-GOG/NTx score, corresponding to an estimated 2.5-point decrease over a 10-year age difference (*p* = 0.018). There was no evidence of an association between FACT-GOG/NTx score and sex, race, ethnicity, neuropathy medication use, diabetes, or disease type.

**Figure 1:**
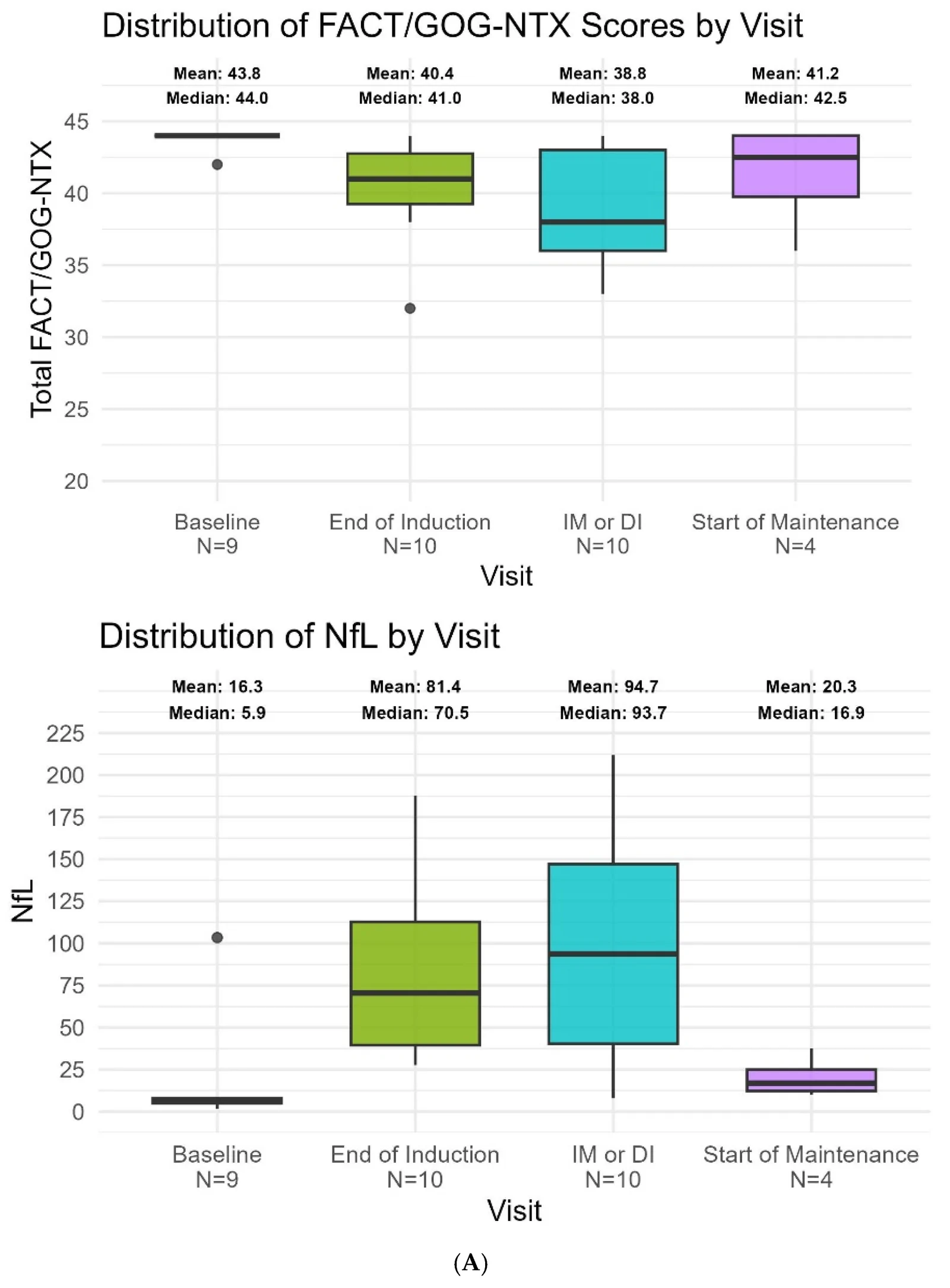

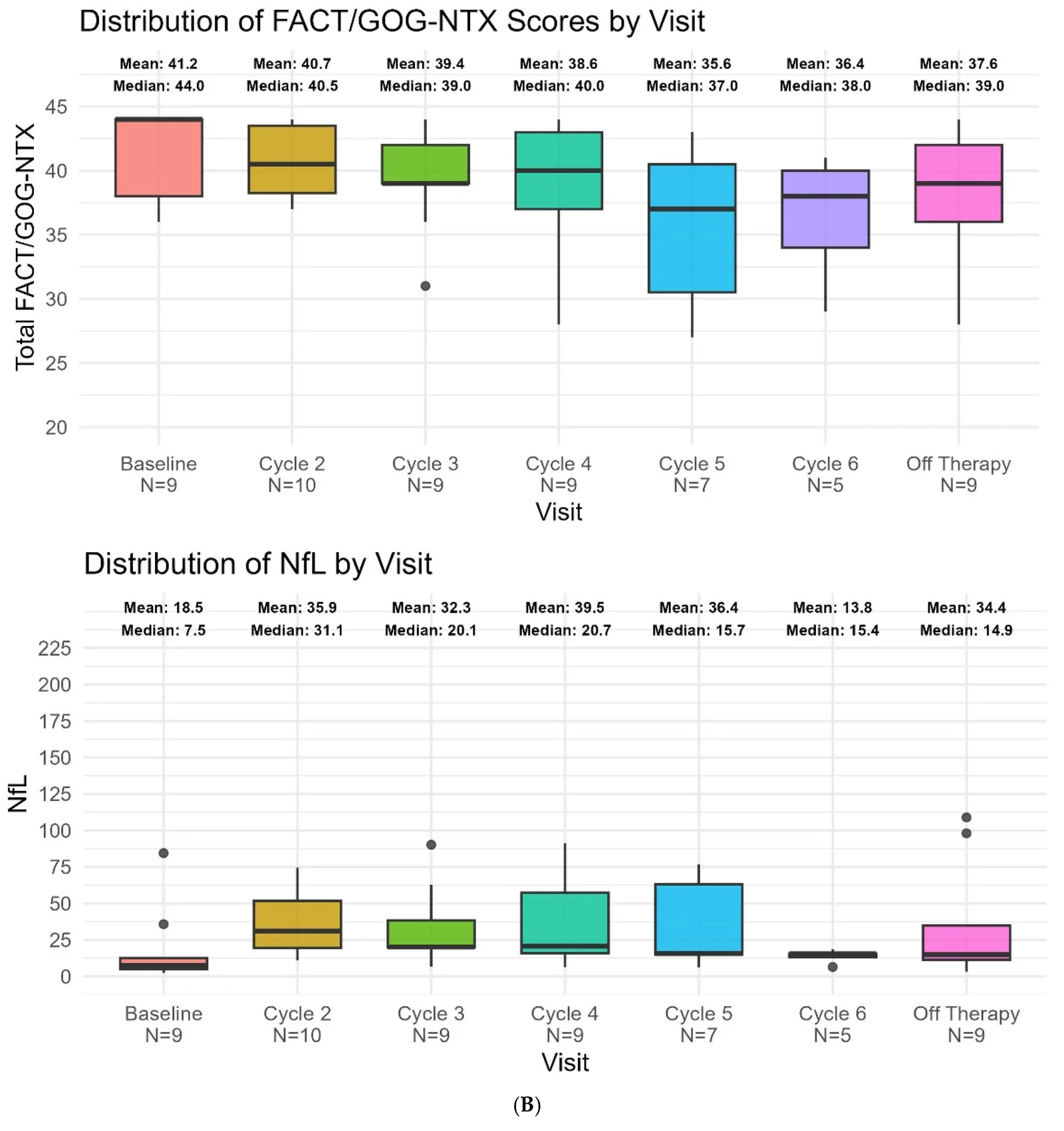
(**A**) Labeled timepoints—leukemia (treated according to Children’s Oncology Group protocols AALL1731, AALL1732, or AALL0434). T0 = baseline; T1 = end of induction; T2 = interim maintenance (IM) or delayed intensification (DI); T3 = start of maintenance. (**B**) Labeled timepoints—Hodgkin lymphoma (treated as per S1826). T0 = baseline; T1 = Cycle 2; T2 = Cycle 3; T3 = Cycle 4; T4 = Cycle 5; T5 = Cycle 6; T6 = Off Therapy.

**Figure 2:**
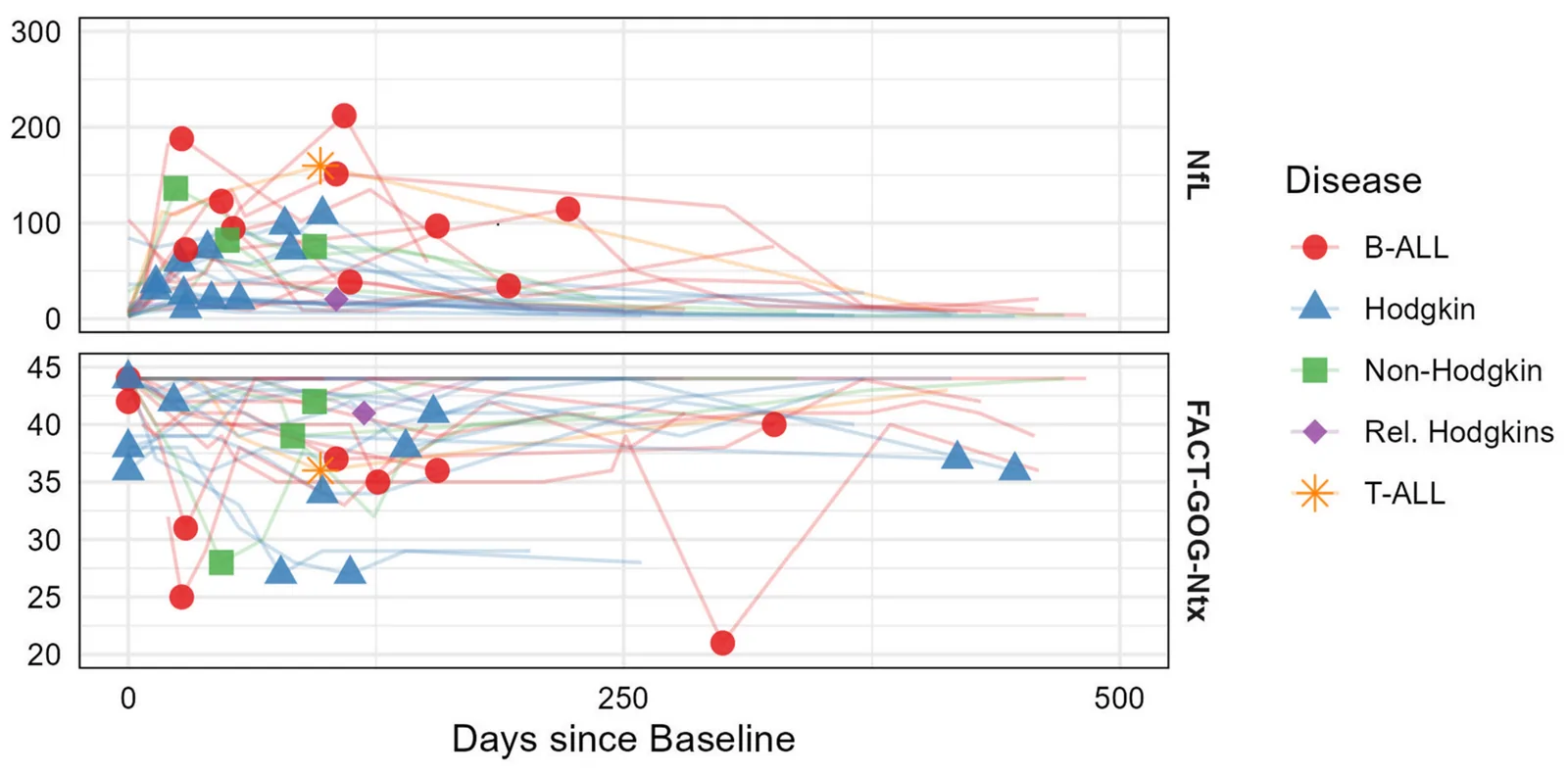
FACT/GOG-NTx and NfL Over Time. Circles represent peak NfL and peak neuropathy.

FACT-GOG/NTx scores declined over time in ALL and HL cohorts, consistent with worsening neuropathy symptoms during treatment (Figure 1A,B). Among patients with leukemia, the lowest median FACT-GOG/NTx scores were observed at T2, corresponding to the end of interim maintenance or beginning of delayed intensification therapy. In the HL cohort, the lowest median FACT-GOG/NTx scores occurred at T4, corresponding to cycle 5 of chemotherapy. These nadirs aligned temporally with periods of cumulative neurotoxic chemotherapy exposure. Similarly, NfL levels increased over the course of therapy in both cohorts and peaked at T2 (leukemia) and T4 (HL), corresponding with the lowest reported FACT-GOG/NTx scores (Figure 2).

### 3.3. Association Between NfL and Neuropathy

Higher NfL levels were significantly associated with worse clinical neuropathy severity as measured by FACT-GOG/NTx scores. After adjusting for age and time, a 50-unit increase in NfL was associated with an average 1.08-point decrease in total FACT-GOG/NTx score, indicating worsening neuropathy symptoms (95% CI: −1.67, −0.49, *p* < 0.001). Similarly, each doubling of NfL was associated with a 0.61-point decrease in FACT-GOG/NTx score (95% CI: −0.90, −0.33, *p* < 0.001).

### 3.4. Neuropathy Risk

Higher NfL levels were strongly associated with increased odds of clinically significant neuropathy. For every 50-unit increase in NfL, the odds of neuropathy were 4.62 times higher (95% CI: 2.11–10.11, *p* < 0.001) (Table 2).

Peak NfL levels were not associated with longitudinal FACT-GOG/NTx scores over time (*p* = 0.275). Sensitivity analyses restricted to patients with predefined study timepoints on day 1 of each chemotherapy cycle demonstrated persistent associations between NfL and FACT-GOG/NTx scores (*p* = 0.001, Table 3). In subgroup analyses, the association between NfL and neuropathy remained significant among patients with leukemia (*p* = 0.003) but was not observed among patients with Hodgkin lymphoma across all timepoints (Table 3).

## 4. Discussion

In this prospective pilot study of children and AYA patients receiving neurotoxic chemotherapy, higher circulating NfL levels were significantly associated with worse patient-reported CIPN severity as measured by the FACT-GOG/NTx. Increasing NfL levels correlated with lower FACT-GOG/NTx scores and higher odds of clinically significant neuropathy, supporting NfL as a promising biomarker of neurotoxicity secondary to axonal damage in pediatric and AYA oncology populations. Importantly, these findings were observed using longitudinal patient-reported neuropathy assessments, suggesting that NfL reflects clinically meaningful symptom burden rather than isolated initial axonal damage.

Our findings add to a growing body of literature supporting NfL as a biomarker of axonal injury in the setting of neurotoxic chemotherapy. Prior adult studies have demonstrated associations between elevated NfL levels and the development of chemotherapy-induced peripheral neuropathy (CIPN) among patients receiving taxanes and platinum agents [28]. However, pediatric and AYA data with vinca alkaloids remain limited despite the high prevalence and long-term functional consequences of CIPN in younger populations.

Our findings are consistent with a recent pediatric study by Pontoppidan et al., which demonstrated progressive increases in plasma NfL levels during vincristine-containing induction therapy in children with ALL using CTCAE-based neuropathy assessment [18]. Importantly, our study extends these observations in several meaningful ways. Unlike the prior study, which relied on clinician-reported CTCAE grading and included only leukemia patients, our analysis incorporated validated longitudinal patient-reported FACT-GOG/NTx assessments across both leukemia and lymphoma populations. Given the well-described limitations of CTCAE in detecting mild and early neuropathy symptoms, the use of longitudinal PROs in our study likely provided a more sensitive and clinically relevant measure of neuropathy burden and better captured the patient experience of CIPN [7,18].

Interestingly, prior pediatric work has suggested that younger children may demonstrate higher plasma NfL levels during vincristine therapy, potentially reflecting developmental differences in baseline NfL levels or age-related differences in vincristine pharmacokinetics and dosing practices, including dose capping [17,18]. In contrast, our study found that increasing age was associated with worse patient-reported neuropathy severity, and older patients had higher NfL levels. These differing findings are likely to reflect the distinction between biochemical evidence of axonal injury and patient-reported symptom burden and further highlight the complexity of CIPN assessment across pediatric age groups.

In contrast to adult studies, our findings demonstrate that NfL appeared to function primarily as a marker of current CIPN rather than a predictor of future neuropathy severity [29,30,31,32]. While higher NfL levels were associated with worse FACT-GOG/NTx scores at the same timepoint, neither peak NfL levels nor NfL levels measured at the previous study visit were associated with neuropathy severity at subsequent timepoints. Similarly, NfL levels at earlier treatment phases did not predict later FACT-GOG/NTx scores in either leukemia or lymphoma cohorts. These findings suggest that NfL may be most useful as a real-time clinical biomarker of ongoing axonal injury rather than a prognostic biomarker for delayed neuropathy development. This distinction is clinically important, as a biomarker capable of dynamically reflecting active neurotoxicity could support treatment monitoring, toxicity surveillance, and future intervention studies aimed at mitigating progressive nerve injury during therapy. This may be especially important in younger children, particularly patients <5 years of age, where there are currently no validated patient-reported CIPN measures and neuropathy can be difficult to recognize clinically [8,33]. While tools such as the pediatric modified Total Neuropathy Score (ped-mTNS) are available for older children, they remain time-intensive, require trained personnel, and depend on detailed neurologic examination and patient cooperation, which can limit feasibility in routine clinical care in younger patients [33,34,35]. These limitations highlight the potential value of an objective, minimally invasive biomarker such as NfL in pediatric oncology populations.

This study has several limitations. As a pilot study, the sample size was small and included heterogeneous disease populations and treatment regimens, limiting power for subgroup analyses and restricting generalizability. The timing of assessments differed between leukemia and lymphoma cohorts based on treatment structure, and not all patients completed evaluations at identical timepoints. Additionally, while the FACT-GOG/NTx has demonstrated utility in pediatric populations down to age 5, younger children may still have difficulty reporting neuropathy symptoms consistently. Finally, because this was an observational biomarker study, causal relationships between NfL elevations and neuropathy progression cannot be established.

## 5. Conclusions

Despite these limitations, this study provides preliminary evidence supporting NfL as a biomarker of axonal injury associated with CIPN in children and AYAs receiving cancer therapy. The longitudinal design, repeated biomarker assessments, and incorporation of a PRO measure strengthen these findings. Larger multicenter studies are needed to validate these results, define clinically meaningful NfL thresholds, better characterize the timing of NfL changes during therapy, and determine whether NfL can identify patients at risk for clinically significant neuropathy before symptoms become severe. Future work should also evaluate the predictive utility of NfL, including whether early changes in NfL can identify patients most likely to develop severe CIPN, require chemotherapy dose modifications, or experience persistent neuropathy after therapy. Earlier detection, longitudinal monitoring, and eventual risk prediction could create opportunities for timely interventions, including supportive care strategies, rehabilitation services, or chemotherapy modifications aimed at preventing irreversible nerve injury. If validated, NfL could provide an objective tool to complement symptom reporting, facilitate earlier recognition of neurotoxicity, and support more individualized approaches to CIPN monitoring and management.

## Figures and Tables

**Table 1:** Subject characteristics.

Characteristic	*N* = 26
Age at Diagnosis, *Median (Q1, Q3)*	15.4 (9.4, 17.0)
Sex	
Female	11 (42%)
Male	15 (58%)
Race	
White	22 (85%)
Black/African American	1 (3.8%)
Asian	2 (7.7%)
American Indian/Alaska Native	1 (3.8%)
Ethnicity	
Non-Hispanic/Latino	23 (88%)
Hispanic/Latino	3 (12%)
Disease Type	
Leukemia	11 (42%)
Lymphoma	15 (58%)
Disease Subtype	
Acute Lymphoblastic Leukemia	11 (42%)
Hodgkin	12 (46%)
Non-Hodgkin	3 (12%)
Neuropathy Medications ^^^	
Yes	5 (19%)
No	21 (81%)
Diabetes	
Yes	2 (7.7%)
No	24
Collected Samples, *Median (Q1, Q3)*	8 (7,9)
Baseline FACT/GOG-NTx total ^1^, *Median (Q1, Q3)*	44.00 (43.00, 44.00)
Baseline NfL ^1^, *Median (Q1, Q3)*	6 (3, 10)
Peak NfL, *Median (Q1, Q3)*	74 (34, 114)

^1^ *N* = 1 subject was missing baseline FACT/GOG-NTx score, and *N* = 1 subject was missing baseline NfL measurement. ^^^ = duloxetine or gabapentin used for treatment of chemotherapy-induced peripheral neuropathy.

**Table 2:** Logistic mixed model for the effect of NfL on neuropathy status over time.

Parameter	Time-Adjusted Odds Ratio Estimate (95% Confidence Interval)	Adjusted Odds Ratio Estimate (95% Confidence Interval)	*p*-Value
NfL (50 Units)	4.40 (2.01, 9.64)	4.62 (2.11, 10.11)	<0.001 *

Time-adjusted model includes time since baseline. Adjusted model includes age at diagnosis and time since baseline. 26 subjects, 240 observations; 94 (39.2%) observations were neuropathy positive. * *p* < 0.05.

**Table 3:** Effect of NfL on FACT/GOG-NTx score over time: sensitivity analyses restricted to study timepoints only.

	Entire Sample, 23 Subjects, 105 Observations			Leukemia, 11 Subjects, 40 Observations			Lymphoma, 10 Subjects, 58 Observations		
Covariate	Estimate	95% CI	*p*-Value	Estimate	95% CI	*p*-Value	Estimate	95% CI	*p*-Value
NfL (50 Units)	−1.44	(−2.26, −0.63)	0.001 *	−1.28	(−2.07, −0.49)	0.003 *	−0.32	(−2.65, 2.00)	0.787

Models were adjusted for age at diagnosis and time since baseline. * *p* < 0.05.

## Data Availability

The data presented in this study are available upon request from the corresponding author due to the inclusion of personal health information.

## Supplementary Materials

The following supporting information can be downloaded at: https://www.mdpi.com/article/10.3390/cancers18172756/s1, Table S1: Vinca alkaloid dosing per protocol backbone.
