# Supplementary material for: Neurofilament Light Chain: A Potential Biomarker for Chemotherapy-Induced Peripheral Neuropathy in Pediatric and Adolescent Young Adults with Leukemia or Lymphoma
Source: Cancers (Basel). 2026 Aug 25;18(17):2756. doi: 10.3390/cancers18172756 (PMC13565587; doi:10.3390/cancers18172756)
Supplement: Supplementary file 1 [file cancers-18-02756-s001.zip › cancers-4461615-supplementary.pdf]

Supplemental Material:

Supplemental Table S1. Vinca alkaloid dosing per protocol backbone.

Protocol	Study timepoint	Planned cumulative vinca alkaloid exposure*
**AALL1732 Arm D**	End of Induction	**Vincristine 6 mg/m²**
	Start of Delayed Intensification	**Vincristine 18 mg/m²**
	Start of Maintenance	**Vincristine 33 mg/m²**
**AALL1731 Arms D**	End of Induction	**Vincristine 6 mg/m²**
	Start of Delayed Intensification	**Vincristine 18 mg/m²**
	Start of Maintenance	**Vincristine 30 mg/m²**
**S1826**	After Cycle 2	**Vinblastine 24 mg/m²**
	After Cycle 4	**Vinblastine 48 mg/m²**
	End of Therapy	**Vinblastine 72 mg/m²**

Footnote :Planned cumulative vinca alkaloid exposure is based on protocol-specified dosing and does not account for maximum dose caps, dose reductions, omissions, or other treatment modifications. Vincristine is administered at 1.5 mg/m²/dose (maximum 2 mg/dose) on AALL1731 and AALL1732.
